# Investigating Body Mass Index and Body Composition in Patients with Schizophrenia: A Case-Control Study

**DOI:** 10.1155/2022/1381542

**Published:** 2022-02-27

**Authors:** M. Marthoenis, M. Martina, Rudi Alfiandi, D. Dahniar, Rini Asnurianti, Hasmila Sari, Jacqueline Nassimbwa, S. M. Yasir Arafat

**Affiliations:** ^1^Department of Psychiatry and Mental Health Nursing, Universitas Syiah Kuala, Banda Aceh, Indonesia 23111; ^2^Health Plus Development Communication, Kampala, Uganda; ^3^Department of Psychiatry, Enam Medical College and Hospital, Dhaka 1340, Bangladesh

## Abstract

**Background:**

Antipsychotics exert metabolic side effects, and prolonged treatment with antipsychotics causes changes in body weight and muscle composition. Nevertheless, reports on the changes in body composition of patients with schizophrenia have been limited. This study is aimed at comparing the body mass index and body composition of patients with schizophrenia with healthy individuals in Indonesia.

**Methods:**

A total of 195 patients with schizophrenia (148 males and 47 females) and 195 healthy individuals matched by gender were recruited. Using the Bioelectrical Impedance Analysis method, the participants' body compositions were measured.

**Results:**

Compared to healthy individuals, the patient group exhibited a higher rate of underweight as well as a lower rate of overweight and obesity. Multiple regression analysis confirmed the associations between the body mass index and all measured body compositions. Furthermore, the diagnosis of schizophrenia is significantly associated with lower muscle mass, lower bone mass, higher basal metabolic rate, older metabolic age, and higher total body water.

**Conclusions:**

The results showed that patients with schizophrenia are at a greater risk of a lower quality of certain components of body composition. Priority should be given to research that addresses increasing the patient's level of physical activity.

## 1. Introduction

Obesity is more prevalent in patients with schizophrenia than in the general population [1, 2], even after controlling for age, gender, and psychiatric practice attended [3]. Obesity is also an independent risk factor for multiple chronic conditions, including cardiovascular diseases, diabetes, hypertension, and stroke [4–6]. Among patients with schizophrenia, the significant predictors of obesity include gender, education level, smoking behavior, type 2 diabetes, a higher level of triglycerides [7], and antipsychotic medication [8].While a patient's body mass index (BMI) is a marker for their nutritional status, it does not reflect the changes in their body composition. The consideration of detail of body composition is significant because, although BMI is significantly correlated with fat mass, the value can be misleading depending on the individual level of adiposity [9].

In recent years, the Bioelectrical Impedance Analysis (BIA) method has been more frequently used to measure body composition variables. BIA is a better indicator of obesity than BMI in patients with schizophrenia [10]. It works with the principle that the transit time of a low-voltage electric current through the body depends on the characteristics of individual body composition [11]. While the dual-emission X-ray absorptiometry (DXA) is the contemporary reference method for the body composition assessment, as it has proven more accurate body composition components [12, 13], accessibility to this sophisticated measure is not always feasible. Using portable BIA equipment is preferable in this study because it is invasive, inexpensive, relatively quick to operate, and suitable for examining a large number of subjects.

Several studies using the BIA have been conducted measuring the body composition of patients with schizophrenia. A hospital-based study found that male patients with schizophrenia under antipsychotic treatments have more body fat, a higher percentage of fat, lower muscle mass, and lower body water compared to the healthy controls, while the female patients have a significantly lower percentage of body fat, higher muscle mass, and higher body water compared to the health controls [9]. Furthermore, a community-based survey found that individuals with schizophrenia tend to have abdominal obesity, higher fat percentage, and lower muscle mass [14]. Another study found that the body fat was associated with height, weight, BMI, and abdominal and waist circumference, while the basal metabolic rate was associated with age, height, weight, abdominal, and waist circumference among patients with schizophrenia [15]. The inconsistency of factors associated with components of body composition between studies demands further investigations.

While BMI studies have been conducted among patients with mental health disorders in Indonesia [16–18], body composition studies have not been conducted. The proximation of obesity or body fat among patients with schizophrenia in the country may therefore be erroneous with available data on BMI, and management protocols may not be well informed. The main objective of this study was to measure BMI and body composition (using BIA) among psychiatric inpatients with schizophrenia and compare them with healthy controls. To the best of our knowledge, this is the first targeted study ever conducted in Indonesia. Based on the findings of previous related studies and theories, we hypothesize that the patients with schizophrenia have higher BMI, total body water, and body fat but lower muscle mass, bone mass, physique rating, and basal metabolic rate compared to the healthy controls.

## 2. Methods

### 2.1. Study Subjects

The subjects were 195 inpatients (148 males and 47 females) diagnosed with schizophrenia based on DSM-IV diagnostic criteria at the Aceh Psychiatric Hospital in Indonesia. The subjects' diagnoses were obtained from their medical records. As a reference group, 195 healthy volunteers were also included. The reference group was recruited from the general population using convenience sampling methods. Before inclusion into the study, the volunteers were screened with a semistructured interview to ensure the absence of both psychiatric disorders and the use of antipsychotics. The semistructured interview consists of questions whether they had ever been diagnosed with any mental disorder or had the history of antipsychotic medication use or think they had experienced any psychiatric symptoms. Only volunteers who confirm that they never had any of those problem were included in the study. For both the patients and volunteers, we also asked about their age, marital status, education, and occupation. The financial status was examined by their occupation status, whether they had bank account and savings, either at the bank or elsewhere. Data collection was conducted between June and August 2020. The study was approved by an ethics committee at Universitas Syiah Kuala, and all participants provided informed consent before participating in the study. Permit for data collection among patients with schizophrenia was also obtained from the psychiatric hospital management.

### 2.2. Procedure

The subjects' demographic information and medications were compiled from their medical records, and the patients' antipsychotic medication was converted to a chlorpromazine equivalent [19]. Body composition was measured with the Tanita RD-953 analyzer [20]. This measurement procedure requires the subject to stand barefooted on the analyzer, while the height, date of birth, and level of activity were entered into the device. The device then calculates their weight, BMI, body fat percentage, muscle mass, muscle quality score, physique rating, bone mass, visceral fat, basal metabolic rate, metabolic age, and total body water. In addition, the blood pressure was measured using electronic blood pressure equipment while in a sitting position.

The body fat percentage was defined as the proportion of fat to the total body weight. Muscle mass is the predicted weight of muscle in the body, which includes the skeletal muscle, smooth muscles, and water contained in the muscles. Muscle quality score is calculated by the amount of muscle mass against the individual height. The larger score, the more muscle mass an individual has. Physique rating measures the muscle and body fat level and is rated as one of nine body types. Bone mass predicts the weight of bone minerals in the body. Visceral fat explains the amount of fat in the abdominal area to protect the vital organs. Basal metabolic rate is the daily minimum level of energy or calories that the body needs when at rest to function effectively. The metabolic age is the comparison of individual basal metabolic rate to the average basal metabolic rate of the chronological age group. Total body water is the amount of fluid in the individual body [21]. Furthermore, BMI was categorized as the following: underweight (BMI < 18.5), normal (18.5 ≥ BMI < 24.9), overweight (25 ≥ BMI < 27), and obese (BMI ≥ 27).

### 2.3. Statistical Analysis

Demographic and clinical characteristics were analyzed using descriptive statistics indicating the number and percentage, mean and standard deviation (SD), or median and interquartile range (IQR). The differences in demographic and clinical variables between the patients and the control group were performed using the Chi-squared test for categorical data and independent sample *t*-test analyses for continuous data. The independent sample *t*-test was also used to calculate the mean differences in body composition between the groups. Furthermore, multiple linear regression analysis was performed to predict demographic and other variables that are independently associated with each component of body composition. The independent variables associated with the components of body composition were included in the multiple linear regression analysis. Only variables with a significant *p* value (<0.05) were reported. Data analysis was carried out using the STATA 13 statistical software [22].

## 3. Results

### 3.1. Demographics

The mean (SD) age of all participants was 34 years (7.5), and the patients with schizophrenia were older, on average (36 years) (0.6), than the control group (32 years) (0.6) (*p* < 0.001). More than half (63.8%) of the control group was married, while only approximately one-third (36.2%) of the patients were married (*p* < 0.001). The vast majority of the patients only graduated from elementary school (98.3%), whereas the majority of the control group had a university education level (95.2%). Furthermore, the vast majority of the patient's group were jobless or not working at the moment (80%), did not have bank account (97.4%), and did not have any savings, either at the bank or somewhere else (86%), indicating a low socioeconomic status of the patient group. No difference in systolic and diastolic blood pressures was observed. The median dosage of medication converted to a chlorpromazine equivalent was 475 mg per day (IQR = 400-700). Detailed sociodemographic and clinical characteristics of the study participants are presented in Table 1.

### 3.2. Body Composition Measurements

Table 2 shows the differences in body composition between patients with schizophrenia and healthy controls. Overall, the patients with schizophrenia have a lower mean of body composition components compared to the healthy controls, except for metabolic age and total body water. The patients were shorter in height and lower in weight, BMI, muscle mass, bone mass, and basal metabolic rate (*p* < 0.001) compared to the healthy controls. In addition, their mean metabolic age was older (*p* = 0.03), and their total body water was higher than the control group (*p* < 0.001). A mean difference of body fat, muscle quality score, physique rating, and visceral fat between the groups was not observed (*p* > 0.05). The proportion of BMI categories between groups was also significantly different (*p* < 0.001). The patients' group has a higher underweight proportion but less prevalence in the overweight and obese groups. The details of the difference in BMI between patients and healthy controls are presented in Table 3.

### 3.3. Multiple Regression Analysis

Further statistical analysis confirms that BMI was associated with all measured body composition factors. BMI was associated with a higher body fat percentage, heavier muscle mass, better muscle quality, lower physique rating, higher bone mass, a larger amount of visceral fat, higher basal metabolic rate, older metabolic age, and lower total body water. Similar to BMI, gender was also associated with all components of the body composition, except with muscle quality and physique rating. Furthermore, schizophrenia was independently and significantly associated with lighter muscle mass, lower bone mass, higher basal metabolic rate, older metabolic age, and more total body water. Regular exercise was also significantly associated with a lower percentage of body fat, heavier muscle mass, better muscle quality, better physique rating, heavier bone mass, and lower visceral fat. Detailed regression analyses examining the association between the diagnosis of schizophrenia under antipsychotic treatments and body composition are presented in Table 4.

## 4. Discussion

This study examined the BMI and body composition of patients with schizophrenia compared with healthy controls. Patients undergoing antipsychotic treatment have a lower mean of muscle mass, bone mass, and basal metabolic rate while also exhibiting an older metabolic age and a higher percentage of body water compared to healthy subjects. Some of the findings in this study are in line with the previous reports, whereas others contradict the literature.

Previous studies have indicated that body fat mass and body fat percentage were higher in patients with schizophrenia than healthy individuals [9, 23]. In contrast, this study suggests that both groups have an average body fat of 25 kg, and no statistical difference was found between the groups. The lower muscle mass in the patients' group found in this study is in line with an earlier report [9], which stated that these findings are associated with several factors, including age and low physical activity [24]. Therefore, our findings are relevant to the treatment condition of the study population, as the vast majority of patients were locked in nursing wards and barely had any physical activity.

A relatively higher rate of underweight and a lower rate of overweight and obesity among the patient group compared to the normal controls were found in this study. The finding of 10.3% of patients with underweight has decreased from the 17% underweight rate reported in a previous study in the same setting [18], but it is still relatively higher than the rate of underweight in patients with schizophrenia reported in a recent meta-analysis (6.2%) [25]. Nevertheless, the rate of overweight and obesity among the patients has changed from 8% and 5% reported earlier [18] to 7.7% and 18.5% found in this study, respectively. A lower rate of overweight and obese among inpatients with schizophrenia might be because of the majority of the patients come from low socioeconomic groups with financial difficulties. In other words, the patients had been poor and could not afford adequate food before hospitalization. During hospital treatment, the patients only eat food provided by the hospital and barely any snacks in between. Therefore, their body weight was most likely to be within normal weight. Also, the financial status of the patients, where the fast majority did not have any occupation, had no bank account, and had no savings, confirms their low socioeconomic status. Furthermore, following our earlier study dissemination, the psychiatric hospital management has made several changes concerning the meal and nutritional management of the patients. These changes include the employment of new nutritionists and the replacement of the food supplier, thus providing better services. The improvement of the prehospital financial condition of the patients might also be responsible for the higher rate of obesity during hospitalization.

The correlation between BMI and all body composition components measured is consistent with previous reports [9, 23, 26]. Despite BMI being a marker for nutritional status in patients with schizophrenia, it may not reflect changes in body composition. Positive correlations between BMI score and body fat percentage and visceral fat score confirm that as the BMI increases, the other two variables also increase. The increased BMI in patients with schizophrenia is the consequence of antipsychotic medications, psychosocial and socioeconomic risk factors, and an unhealthy lifestyle [27]. Behavioral interventions that control the nutritional intake and encourage regular exercise are proposed, along with other relevant interventions during hospitalization. As such, this study also highlights the importance of regular exercise for improving the body composition of the study subjects.

A significant decrease in bone mass among the patients' group was found. This is associated with female gender, irregular or no exercise, lower BMI, and the diagnosis of schizophrenia. Low bone mineral density is common in patients with schizophrenia [28, 29], and antipsychotic medication is one of the risk factors [29]. However, reports have also indicated that long-term clozapine may protect bone density [30, 31]. Regular exercise also increases bone mass in female adolescents [32]. Further studies might consider whether providing more physical activity and other possible interventions could improve patients' bone mineral density.

This study has several limitations. First, it was conducted during the COVID-19 outbreak. Due to the social distancing policy implemented by the local government, a limited number of patients and healthy controls were included in the study. Second, only hospitalized patients were included, leaving outpatients or those living in the community but using antipsychotics unexamined. Third, several important information such as the age of onset, duration of illness, symptom severity, functional level, and medication was not collected from the patients. Last, the reference group was only matched with gender and not age. Future researchers might consider collecting the abovementioned information from the patients, matching for both age and gender, and including outpatients with schizophrenia or those living in the community in their studies.

## 5. Conclusion

This study highlighted several issues. First, cases of underweight still prevail in this population, despite the rate being lower than previously reported. Second, the diagnosis of schizophrenia is associated with poorer body composition compared to healthy subjects. Lastly, physical activity should be proposed to patients as part of daily treatment during hospitalization.

## Figures and Tables

**Table 1:** Sociodemographic and clinical characteristics of participants (*n* = 390).

Characteristics	Total	Schizophrenia	Control	*x*2 or *t*	*p* value
Age (years old)	34 ± 7.5	36 ± 0.6	32 ± 0.6	4.8	0.001^∗^
Marital status (%)				36	0.001^∗^
Unmarried	177 (45.4)	118 (66.7)	59 (33.3)		
Married	213 (54.6)	77 (36.2)	136 (63.8)		
Education				232.6	0.001^∗^
Elementary (1-6 yrs)	117 (30.0)	115 (98.3)	2 (1.7)		
High school (7-12 yrs)	126 (32.3)	73 (57.9)	53 (42.1)		
University	147 (37.7)	7 (4.8)	140 (95.2)		
Occupation				199.2	0.001^∗^
Jobless or not working	95 (24.4)	76 (80)	19 (20)		
Irregular job	161 (41.3)	118 (73.3)	43 (26.7)		
Formal job	134 (34.4)	1 (0.7)	133 (99.3)		
Having bank account				343.5	0.001^∗^
Yes	197 (50.5)	7 (3.6)	190 (96.4)		
No	193 (49.5)	188 (97.4)	5 (2.6)		
Having savings (at the bank or elsewhere)				267.7	0.001^∗^
Yes	168 (43.1)	4 (2.4)	164(97.6)		
No	222 (56.9)	191 (86)	31 (14)		
Systolic blood pressure (mmHg)	121 ± 52	120 ± 73	122 ± 11	0.36	0.71
Diastolic blood pressure (mmHg)	84 ± 11	84 ± 13	84 ± 9	0.45	0.65

**Table 2:** The difference in body composition between the patients and healthy controls.

Body composition	Range score (unit)	Schizophrenia	Control	*t*-test	*p* value
		Mean (SD)	Mean (SD)		
Height	cm	161 (8.2)	166 (6.2)	6.23	0.001
Weight	kg	60.6 (11.9)	68.8 (12.7)	6.61	0.001
Body mass index	kg/m^2^	23.2 (4.9)	25.1 (4.2)	4.19	0.001
Body fat percentage	%	25.1 (10.3)	25.5 (9.9)	0.4	0.68
Muscle mass	kg	42.7 (6.7)	47.5 (9.1)	5.9	0.001
Muscle quality score	Point	64.8 (12.9)	65.6 (13.1)	0.6	0.54
Physique rating	1-9^∗^	3.6 (2.9)	4.1 (1.6)	1.5	0.11
Bone mass	kg	2.4 (0.3)	2.7 (0.4)	7.6	0.001
Visceral fat rating	1-59^∗^	7.6 (4)	8.1 (4)	1.2	0.2
Basal metabolic rate	kcal	1285 (190)	1461 (244)	7.91	0.001
Metabolic age	Year	38 (11)	36 (9)	2.1	0.03
Total body water	%	50.5 (6.8)	47.7 (5.7)	4.2	0.001

**Table 3:** The difference in body mass index between patients and healthy control group.

Body mass index	Total	Schizophrenia	Control	*x*2	*p* value
	*n* (%)	*n* (%)	*n* (%)		
Underweight	29 (7.4)	20 (10.3)	9 (4.6)	< 0.001	29.85
Normal	212 (54.4)	124 (63.5)	88 (45.1)		
Overweight	63 (16.2)	15 (7.7)	48 (24.6)		
Obese	86 (22)	36 (18.5)	50 (25.7)		

**Table 4:** Multiple regression analysis for the detailed body composition.

Variable	*β*-Coefficient	*p* value
Body fat		
Gender	-10.16	<0.001
Regular exercise	-2.2	0.018
BMI	1.32	<0.001
Muscle mass		
Gender	11.16	<0.001
Regular exercise	2.47	0.004
Schizophrenia	-3.39	<0.001
BMI	0.73	<0.001
Muscle quality		
Age	-0.18	0.031
Regular exercise	5.43	0.003
BMI	0.29	0.034
Physique rating		
Regular exercise	0.92	0.029
BMI	-0.13	<0.001
Bone mass		
Gender	0.49	<0.001
Regular exercise	0.10	0.038
Schizophrenia	-0.2	<0.001
BMI	0.04	<0.001
Visceral fat		
Age	0.11	<0.001
Gender	2.73	<0.001
Regular exercise	-0.71	0.027
Weight	0.07	<0.001
BMI	0.52	<0.001
Basal metabolic rate		
Gender	250.97	<0.001
Schizophrenia	-26.14	<0.001
BMI	25.63	<0.001
Metabolic age		
Age	0.68	<0.001
Gender	-6.01	<0.001
Schizophrenia	1.83	0.011
BMI	1.05	<0.001
Total body water		
Gender	4.29	<0.001
BMI	-0.69	<0.001
Schizophrenia	1.47	0.005

## Data Availability

The data that support the findings of this study are available on request from the corresponding author.
